# Approach to Decompensated Right Heart Failure in the Acute Setting

**DOI:** 10.3390/jcm13030869

**Published:** 2024-02-02

**Authors:** Catherine V. Levitt, Caitlin A. Williams, Jalil Ahari, Ali Pourmand

**Affiliations:** 1 Department of Emergency Medicine, The George Washington University School of Medicine and Health Sciences, Washington, DC 20037, USAcawilliams@gwu.edu (C.A.W.); 2 Pulmonary and Critical Care Medicine, The George Washington University School of Medicine and Health Sciences, Washington, DC 20037, USA

**Keywords:** acute right heart failure, right ventricular failure, pulmonary vascular resistance, pulmonary arterial hypertension

## Abstract

Acute right heart failure (ARHF) arises when the right ventricle fails to pump blood efficiently to the pulmonary circulation. This inefficiency leads to a decreased blood supply to various organs. ARHF is a significant health concern, often leading to increased hospital admissions and being associated with a higher risk of mortality. This condition underscores the importance of effective cardiac care and timely intervention to manage its complications and improve patient outcomes. Diagnosing ARHF involves a comprehensive approach that includes a physical examination to evaluate the patient’s fluid status and heart-lung function, blood tests to identify potential triggers and help forecast patient outcomes and various imaging techniques. These imaging techniques include electrocardiograms, point-of-care ultrasounds, computed tomography, cardiac magnetic resonance imaging, and other advanced monitoring methods. These diagnostic tools collectively aid in a detailed assessment of the patient’s cardiac and pulmonary health, essential for effective management of ARHF. The management of ARHF focuses on addressing the underlying causes, regulating fluid balance, and enhancing cardiac function through pharmacological treatments or mechanical support aimed at boosting right heart performance. This management strategy includes the use of medications that modulate preload, afterload, and inotropy; vasopressors; anti-arrhythmic drugs; ensuring proper oxygenation and ventilation; and the utilization of heart and lung assist devices as a bridge to potential transplantation. This review article is dedicated to exploring the pathophysiology of ARHF, examining its associated morbidity and mortality, evaluating the various diagnostic tools available, and discussing the diverse treatment modalities. The article seeks to provide a comprehensive understanding of ARHF, its impact on health, and the current strategies for its management.

## 1. Introduction

Acute right heart failure (ARHF), otherwise known as right ventricular (RV) failure, results when the RV is unable to effectively pump blood through the pulmonary circulation [1,2,3,4]. ARHF is defined by the World Health Organization as reduced blood supply to other organs and/or clinical signs of venous congestion with fluid retention, resulting from RV failure [1]. This is thought to be the result of a combination of mechanisms—excessive preload; excessive afterload; reduced inotropy (contractility); and reduced lusitropy (relaxation) [2,3,4,5,6]. ARHF can have many inciting incidents that impact the hemodynamics of the cardiopulmonary circulation. These include, but are not limited to, infections/sepsis, arrhythmias like supraventricular tachycardia (SVT), anemia, pregnancy, medication withdrawal, and acute cardiopulmonary events such as myocardial infarction (MI) or pulmonary embolism (PE) [3,4,7,8]. These etiologies are frequently seen in the emergency department; however, the management of patients with ARHF who present to the emergency department remains challenging. Treatment is based on treating these inciting factors, managing fluid status, and improving cardiac function via medications or mechanical support to help augment the function of the right heart as a means to avoid an urgent transplant, which some patients ultimately do require as their definitive treatment. This article aims to provide a review of the different strategies for managing these patients for the emergency physician.

## 2. Methods

We performed a narrative literature review, searching the PubMed and SCOPUS databases from their inception up to 12 December 2023. This search utilized the Medical Subject Headings (MeSHs) term ‘Heart Failure’. This was further refined through the application of Boolean operators to focus our investigation specifically on “right-sided heart failure”, “right ventricle”, and “decompensated” exploring its underlying pathophysiology, causes, diagnostic approaches, and treatment considerations. Our search encompassed a broad spectrum of study types, including observational studies, case series, case reports, narrative reviews, systematic reviews, and guidelines, with the aim of comprehensive inclusion of relevant literature. We restricted our search results to include articles that specifically involve human adult patients and are presented in the English language (Supplementary Materials).

The search yielded 229 matches. Initially, two independent reviewers screened titles and abstracts, resulting in the inclusion of 42 articles in this review. Additionally, the authors exercised discretion in adding further references. Articles were excluded if they were not primarily in English or lacked an English translation, focused on pediatric patients, or presented preliminary/unpublished results. Any discrepancies in the selection of eligible articles were resolved through discussion among the investigators. It is important to note that this study is derived from previously conducted research and does not involve data from human participants or animals performed by any of the authors.

## 3. Results

### 3.1. Physiology and Pathophysiology

The right heart is distinct from the left heart, with the right heart’s circulatory system consisting of the right atrium, tricuspid and pulmonary valves, the right ventricle (RV), and pulmonary vasculature [8]. It is known that all causes of left-heart failure will eventually lead to right-heart failure [9]. This is because the right ventricle contains a relatively thin wall (compared to the left ventricle) that is designed to pump into the pulmonary circulation, which normally has low resistance, high compliance, and low impedance [1,10,11,12]. The thin wall of the RV is more dependent on coronary perfusion pressure and is greatly impacted by increases in RV pressure, as well as systemic hypoperfusion [5]. Failure of the left ventricle to handle the preload leads to a progressive increase in pressure in the pulmonary vasculature system, causing gradual hypertrophy of the vascular smooth muscle and fibrotic changes [11]. As a result, pulmonary vascular resistance and RV afterload increase [11]. Over time, RV hypertrophy and eventually RV dilatation may occur [11]. Fibrosis of the RV can also occur with subsequent diastolic dysfunction and resultant right atrial enlargement [1].

The function of the RV is influenced by preload, afterload, contractility, and relaxation. RV preload refers to the end diastolic volume/pressure, or the load prior to contraction [8]. RV afterload is influenced by the pulmonary vascular resistance (PVR) and the compliance of the chamber [8]. Excessive preload can be seen with regurgitant pulmonic or tricuspid valves, venous congestion, or left-to-right intracardiac shunts [2,3,5,8]. Excessive afterload may be due to stenotic valvular diseases (pulmonary or mitral valve), obstructive pulmonary vascular diseases (PE, tumor emboli, etc.), or elevated pulmonary artery pressure secondary to pulmonary hypertension or pulmonary venous congestion [2,3,8]. Pulmonary hypertension (PH) is classified into five distinct groups: group I is characterized by increased pulmonary arterial pressure/resistance (otherwise called pulmonary arterial hypertension (PAH)), group II is caused by left heart disease or congenital heart disease, group III is due to lung disease or hypoxia, group IV is from pulmonary arterial obstruction, and group V is due to unclear or multifactorial mechanisms [4,13,14]. The underlying pathophysiology of each group of PH leads to slightly different right ventricular responses and remodeling; for example, those with congenital heart disease begin to experience remodeling in utero, whereas those with chronic thromboemboli have vascular remodeling related to the location and extent of the obstruction [15]. Several animal models have explored the role of cytokines and molecular changes in the setting of right ventricular overload, chronic RV failure, and ARHF [5].

Reduced RV inotropy is noted to occur due to myocardial ischemia, myocarditis, or other nonischemic cardiomyopathies, systemic sclerosis, and pericardial disease, whereas reduced lusitropy could result from myocardial fibrosis, stiffening, infiltrative cardiomyopathies, or constrictive pericarditis [2,8]. The abnormalities of RV load (preload and afterload) or myocardial function (contractility and relaxation) often occur simultaneously, exacerbating each other in a cycle ultimately leading to acute or chronic right heart failure [2].

Systemic venous congestion is common in RV failure, as the dysfunction leads to impaired RV filling and increased right atrial pressures [3]. As RV failure progresses, the chamber becomes more spherical, leading to tricuspid regurgitation and worsening systemic venous congestion [3,16]. The combination of systemic venous congestion and backward transmission of elevated central venous pressure (CVP) may cause a deleterious effect on other vital organs, including renal dysfunction, a condition known as cardiorenal syndrome (CRS) [11,16]. Increases in CVP and systemic venous congestion are associated with increases in renal vein pressure (RVP), decreases in renal perfusion pressure (RPP), and an increase in renal interstitial pressure. These changes result in renal parenchymal hypoxia, tubular dysfunction, and activation of the renin-angiotensin system [5,11], leading to further increases in intravascular volume expansion and venous congestion, thereby causing worsening of renal and cardiac function [11].

### 3.2. Epidemiology and Mortality

ARHF is suggested to account for 3–9% of all heart failure admissions, and despite being less prevalent than left heart failure, ARHF mortality is estimated to be roughly 6–14% [5,17]. As previously mentioned, pulmonary arterial hypertension (PAH) and left heart failure are two significant etiologies of right heart failure. Studies estimate that approximately 15 million people in the United States have group 1 PAH and that approximately 25% of patients with left heart failure have group 2 PAH [4]. In patients with biventricular failure (both right and left heart failure with reduced ejection fraction), mortality increases by 2.4 fold compared to those with isolated left heart failure [17]. Right heart failure is the leading cause of death for patients with pulmonary arterial hypertension [18], carrying a mortality rate of close to 40% for those who require inpatient intensive care and inotropic support [19]. According to the ECHO-COVID studies, which looked at the echocardiogram characteristics of patients admitted to the intensive care unit (ICU) with the COVID-19 virus, 22.5% of patients had RV dysfunction and 17.5% showed acute cor pulmonale (ACP), and ACP was associated with a significant increase in mortality with an odds ratio of 2.01 for in-hospital mortality [20,21]. In patients whose RV failure is caused by inferior MI, they are at increased risk for shock, arrhythmia, and eventual death, even with revascularization of coronary vessels [5].

RHF can lead to multiorgan failure, which in turn increases patient mortality. As such, it is important to monitor vital organs closely for signs of failure. This includes monitoring global tissue perfusion and oxygenation, renal, hepatic, and central nervous system function, as well as myocardial perfusion and function. In both acute and chronic HF with exacerbations, renal function closely correlates to prognosis [4,11]. Right ventricular failure is present in one-third of patients who present with sepsis and is associated with a 2-3-fold increase in mortality for those in septic shock [14]. In general, indicators of patients nearing death include rising lactate levels, a decrease in mixed or central venous oxygen saturation, and decreasing diuresis [1]. Similarly, elevated BUN and ascites are also associated with long-term mortality [7]. Patients with signs of end-organ dysfunction should be monitored in the ICU [1,4].

### 3.3. Diagnosis and Evaluation

In the emergent setting, it may be difficult to clinically distinguish between acute right or left heart failure; therefore, a detailed history and physical exam and a high level of suspicion can help guide providers. Findings suggestive of ARHF may include dyspnea, anxiety, early satiety, tachycardia, tachypnea, hypoxemia, peripheral edema, relative hypotension, cool/clammy skin, abdominal fullness, or right upper quadrant tenderness [2,3,4,8,9,12]. Physicians should look for evidence of elevated jugular venous pressure, ascites, or hepatojugular reflux, and heart sounds such as tricuspid regurgitation, a loud pulmonary component of the second heart sound (S2), fixed splitting of the S2, or a RV heave [2,4,8,9]. However, it is important to note that physical exam findings, particularly in the early stages, are neither sensitive nor specific, and their absence does not rule out the disease4. Signs of end organ hypoperfusion with an elevated lactate and nonspecific brain natriuretic peptide (BNP and NT-proBNP) in the proper setting are also suggestive of ARHF and should be followed up by evaluations of the kidney and liver function as well [2,4,8,9]. 

Hyponatremia, defined as sodium levels less than 136 mEq/L, is associated with increased morbidity and mortality [4]. Elevated c-reactive protein (CRP) may be a sign of infection or increased inflammation in these patients and is associated with increased mortality [4]. Anemia, even isolated iron-deficiency anemia, has been suggested to worsen RV function, and recommendations include maintaining hemoglobin levels greater than 10 g/dL [10].

An electrocardiogram (EKG) with evidence of right heart pathology such as right atrial dilation, rightward axis deviation, right ventricular hypertrophy, right bundle branch block, or arrhythmogenic right ventricular cardiomyopathy (ARVC) may suggest the diagnosis of ARHF [2,4,8,9,12]. Dysrhythmias may also be noted on the EKG due to rises in right atrial pressures [17].

A definitive diagnosis is obtained via Cardiac Magnetic Resonance Imaging (MRI) [15] and invasive hemodynamic testing (right heart catheterization), the discussion of which is outside the scope of this review. Computed tomography (CT) scans, while not sensitive for diagnosis in early RV failure, can help to determine underlying pulmonary pathologies, including pulmonary embolism, pneumonia, and pulmonary fibrosis [4]. However, point-of-care ultrasound (POCUS) of the heart by the emergency physician can provide additional data, which can be immediately helpful for the diagnosis. All echocardiographic views can help provide the clinician with information on diagnosis and can be used to identify extrinsic causes of acute RV failure, such as pericardial tamponade, a common mimic of ARHF [3]. The apical 4 chamber view can demonstrate right ventricular dilation and enlargement compared to the left ventricle, and when using M-mode aligned with the tricuspid annulus, measurement of the tricuspid annular plane systolic excursion (TAPSE) can be obtained [1,2,3,8,9,12]. TAPSE is a marker of right ventricular systolic function, and when decreased to less than 1.7 cm, it can be associated with ARHF [2,8,9]. In PAH and PE, there may also be evidence of intraventricular septal flattening on this view with a D-shaped LV [4]. Additionally, a plethora of inferior vena cava (diameter < 10 mm) without inspiratory collapse, reduced fractional area change (<35%), and a left shift of the septum with paradoxical movement can also be seen on bedside echo in patients with right heart failure [2,8,9,12]. Fractional area change (FAC) provides an estimate of the right ventricular systolic function [13]. Right ventricular peak systolic strain (RVPSS), which has been associated with mortality in ARHF, can also be calculated using 2D strain software to evaluate the dynamic changes of the RV during systole as seen on echocardiography [4,22]. Additionally, bedside ultrasound can be useful in detecting hypervolemic status by looking for B-lines in lung fields, and the severity of congestion can be calculated by counting the total number of B-lines [23], as well as the fluid status assessment through the measurement of inferior vena cava diameters in the trans-hepatic or subxiphoid view [24]. 

Right atrial pressure is another important prognostic indicator in patients with right heart failure and provides information on a patient’s volume status [25]. One way to estimate right atrial pressure is through the tricuspid E-wave/A-wave ratio on ultrasound; however, it is not always feasible to measure [25]. IVC dilation of greater than 2 cm and respirophasic collapse of less than 40% are also associated with increased right atrial pressures [25]. Another modality gaining traction is 3D echocardiography-derived right atrial volume, where increased values are indicators of increased right atrial pressure [25].

Pulmonary artery catheters (PACs), also known as Swan-Ganz catheters, are inserted percutaneously via a central vein passing through the tricuspid valve into the RV and advanced towards the pulmonary capillary via the pulmonic valve. It can directly measure the pressures of the right atrium, RV, pulmonary artery, pulmonary capillary wedge pressure, and cardiac output. Using these data, one can calculate the pulmonary artery pulsatility index, pulmonary vascular resistance, RV stroke volume, and right stroke work index [1,4,6]. However, studies have had mixed results in terms of improving survival in patients whose management is guided using PACs [26]. The Evaluation Study of Congestive Heart Failure and Pulmonary Artery Catheterization Effectiveness (ESCAPE) trial showed the addition of PAC did not affect overall mortality or hospitalization [26]. However, post-hoc analysis of the ESCAPE trial by different researchers showed that increases in the right ventricular stroke work index from baseline were associated with improved mortality, decreased need for a ventricular assist device, a transplant, or HF hospitalization. As such, more studies need to be performed to definitively determine the benefit of widespread use of PACs.

### 3.4. Management

Treatment of ARHF should be focused on reversing any underlying inciting events, such as hypoxia, ischemia, or sepsis; correcting the right ventricular dysfunction by optimizing preload and afterload; and improving contractility [1,2,7,8,9,13]. As the majority of treatment modalities include mechanical circulatory support, vasopressors, or pulmonary vasodilators, providers should consider admission to the ICU and early transfer to higher levels of care if needed. 

#### 3.4.1. Medical

##### Preload

The overarching thought process for patients in ARHF as it relates to preload is that they are in a state of volume overload, and therefore the administration of fluids is typically not recommended. However, there are some scenarios in which fluids are helpful, such as when patients are in a state of cardiogenic shock due to a pulmonary embolism or have reduced contractility of the right ventricle due to ischemia [1,2]. Small boluses of 200–300 cc of fluid with frequent reassessment may be appropriate [14]. Administering excessive fluid can lead to increased RV free-wall tension, increased oxygen consumption, and ventricular interdependence, where the interventricular septum is displaced toward the LV because of the relatively large volume and pressure in the RV, which leads to decreased LV preload [4].

In cases of suspected excessive preload leading to ARHF, loop diuretics (furosemide, bumetanide, and torsemide) combined with thiazide diuretics to help augment diuresis are the first choice for management [2,3,8,12]. Loop diuretics are particularly useful as they cause natriuresis, creating a net negative water and sodium balance, thereby reducing volume overload [16]. However, while the use of diuretics can help reduce preload, this can also lead to a decrease in cardiac output. As such, close monitoring of intravascular volume and careful management of fluid balance are very important for patients with ARHF [1,4,10]. If hypotension is encountered while diuresing patients, vasopressors should be considered to maintain systemic blood pressure [8]. Vasopressors that help to increase contractility and systemic vascular resistance (SVR) without increasing PVR, such as norepinephrine, are the ideal first-line choice (see vasopressors subheading for further discussion) [2,8].

Diuretics may be necessary despite an apparent rise in creatinine suggestive of an AKI, as this phenomenon is often due to cardiorenal syndrome and treatment requires restoration of renal perfusion [14,16]. In patients with moderate to severe right ventricular dysfunction, treatment with diuretics has been shown to improve renal outcomes, with improvements in glomerular filtration rate (GFR) >25% due to the relief of venous congestion [27]. Despite the improvement in GFR, there have been studies demonstrating a worsening in renal function during diuretic use leading to an initial elevation in creatinine, though this phenomenon is not shown to be associated with worse outcomes in this patient population8.

If diuresis is not sufficiently decongesting the venous system or is contraindicated, ultrafiltration with renal replacement therapy (RRT) is another option to consider [2,8,11,12]. RRT has the benefit of removing medium- and large-size solutes from circulation that may contribute to CRS [11]. Ultrafiltration with RRT is also thought to mediate plasma levels of BNP, norepinephrine, and aldosterone, with symptomatic improvements seen in patients up to 90 days after treatment, including a reduction in readmission rates [11].

##### Afterload

Any insult that increases left atrial pressure, reduces the cross section of the pulmonary vasculature, or causes vasoconstriction will lead to increased right ventricular afterload [13]. A leading cause of ARHF is pulmonary embolism (PE), and roughly 5% of patients with PE will develop cardiogenic shock [9]. In patients with hemodynamic instability due to PE, systemic thrombolytics, percutaneous catheter-directed thrombolysis, or embolectomy are recommended to decrease the afterload and restore RV function [2,3,9]. Hypoxia (namely an oxygen saturation <90%) is a strong vasoconstrictor, causing an increase in PVR and thereby increasing afterload. Therefore, correction of hypoxia is essential [2,8] (See oxygenation and ventilation for further discussion). 

Pulmonary vasodilators are useful in patients with uncontrolled PAH causing RV failure [3,10]. Different drug classes of medications are available to target elevated pulmonary artery pressure, including: calcium channel blockers (nifedipine), prostanoids (epoprostenol, treprostinil, and iloprost), prostacyclin receptor agonists (selexipag), nitric oxide, endothelin receptor antagonists (ERA) (bosentan, ambrisentan, and macitentan), phosphodiesterase-5 (PDE5) inhibitors (sildenafil, tadalafil), and soluble guanylate cyclase stimulators (riociguat) [2,4,10]. The most recent addition to this list is a new class of medication known as activin receptor inhibitors (stotercept) that restores the balance between growth-promoting and growth-inhibiting signaling pathways. This medication is still under review and not available for clinical use. Calcium channel blockers, though frequently used in PAH for their pulmonary vasodilatory effects, should be used with caution in patients with acute RV failure as they can lead to significant hypotension [1,10]. Also of note is that endothelin receptor antagonists are not recommended in the setting of RV failure [4]. It is also worth noting that some patients are already on a pulmonary vasodilator as part of their home regimen, and it is imperative to continue these medications/infusions as withdrawal can lead to a PH crisis and worsen ARHF [1,17].

Pulmonary arterial vasodilators are used with the guidance of PAC; options depend on the severity of the PAH, available routes of administration, the underlying cause, and the presence or absence of contraindications. For instance, in severe PAH, the drug of choice is an intravenous prostanoid infusion [4,8,10,11,13]. In patients who are intubated, inhaled medications such as inhaled epoprostenol and nitric oxide are available [1,3,4,8,19]. In patients with chronic thromboembolic pulmonary hypertension, riociguat could be used. The latter group should be evaluated for possible surgical intervention, and all patients on vasodilator therapy should be closely monitored. While evidence-based treatment strategies are not widely available for treating RV failure in the setting of heart failure with preserved ejection fraction (HFpEF), the role that pulmonary hypertension plays in the development of right ventricular dysfunction in HFpEF should be considered [28,29]. Researchers have started to investigate the effects of some pulmonary vasodilators on RV failure in HFpEF with mixed results, including phosphodiesterase inhibitors, guanylate-cyclase stimulators, and inhaled NO, but have promise for future studies [28,29].

In patients with left-heart failure leading to PH, pulmonary vasodilators are not beneficial and may worsen the clinical condition [2]. To reduce the afterload in patients with ARHF and left heart disease, normalization of left heart pressure and left ventricular function is recommended via systemic vasodilators and volume removal [2,10]. If medical management fails, options such as a left ventricular assist device (LVAD) or veno-arterial extra corporeal membrane oxygenation (VA-ECMO) to bypass the heart may be used as a bridge to transplant [30]. 

##### Inotropic Agents

In patients experiencing a right-sided myocardial infarction (MI), reduced contractility may lead to acute right heart failure. Right ventricular infarction is rare in isolation but can be seen in approximately 50% of inferior MI [31]. A patient presenting with persistent hypotension with increased jugular venous distension in the setting of clear lung fields should raise suspicion for right ventricular infarction [31]. The treatment for RV infarction includes percutaneous coronary intervention or systemic thrombolytics [2,3,9,31]. Patients with RV infarction are preload-dependent; as such, medications that reduce preload, such as nitrates and diuretics, should be used with caution [3]. Patients who do not have an obviously reversible cause of decreased contractility or who are hemodynamically unstable in the case of acute MI may benefit from positive inotropic medications that improve contractility and have the potential to improve RV systolic function [3]. 

Inotropes improve contractility and cardiac output by design, but they also have the risk of triggering or aggravating arrhythmias [3]. As such, practitioners should exercise caution when using inotropic agents in ARHF when patients have pre-existing hyperdynamic left ventricles, which is often associated with decreased preload, for example, in the setting of hypovolemia or sepsis. Furthermore, inotropes, particularly dobutamine, have occasionally been associated with left ventricular outflow tract obstruction, which may acutely worsen the patient’s presentation [32,33].

Dobutamine acts on dopaminergic and adrenergic receptors in a dose-dependent manner, with intermediate dosing (4.0–10.0 mcg/kg/min) leading to an increase in inotropy and chronotropy given its preference for the beta-1 receptor [8]. Dobutamine is one of the preferred inotropes in patients with RV failure because it amplifies myocardial contractility while also reducing both right and left ventricular afterload [1,10]. However, dobutamine has chronotropic properties and can induce tachycardia at higher doses (>10.0 mcg/kg/min), which can be detrimental to diastolic filling time, particularly in patients with PAH, and arrhythmias [4,9,10,34].

Levosimendan has both inotropic and vasodilatory properties and is known to increase contractility of the myocardium without raising intracellular calcium levels by improving sensitization to calcium [4,35,36]. Its efficacy has been well documented in left ventricular failure; however, data are limited for its use in RV failure [36]. There is new evidence that patients with RV failure on levosimendan drips have improvements in ejection fraction and systolic pulmonary artery pressure [35,36]. Compared to dobutamine, levosimendan significantly impacts right ventricular fractional area change (FAC) and (TAPSE) [35]. It also augments diuresis and reduces BNP levels [36].

Milrinone is a phosphodiesterase 3 inhibitor and inotropic vasodilator that can improve contractility and reduce afterload; however, this can also lead to systemic hypotension and a reduction in right coronary perfusion [3,4,8,9]. Inhaled milrinone may have fewer systemic effects as it allows for preferential pulmonary vasodilation without the risk of systemic hypotension [4,8,10]. Furthermore, simultaneous use of milrinone with inhaled nitric oxide has been shown to significantly improve PA pressure compared to either being used alone [4].

##### Vasopressors

Vasopressors work to achieve adequate systemic arterial pressure, thereby maintaining organ perfusion [4]. Ideally, the vasopressor chosen should increase systemic vascular resistance (SVR) without significantly increasing pulmonary vascular resistance (PVR), while also improving RV contractility [4].

Norepinephrine (NE) is mainly an alpha-1 and beta-1 agonist. It increases the systemic blood pressure via vasoconstriction, CO, and stroke volume [1,4,8,10]. NE has the benefit of reducing the PVR/SVR ratio, thereby improving myocardial oxygen delivery [4]. NE is the vasopressor of choice for patients with PH and RV failure [8,19].

Vasopressin, also known as antidiuretic hormone, acts on the vasopressin (V1 and V2) receptors to constrict vascular smooth muscle [1,8,10]. In animal studies, it has been shown to cause pulmonary arterial vasodilation; however, in humans, the effects are inconclusive [8,10]. However, it has been used in both pediatric and adult settings as a rescue agent for pulmonary hypertension and RV failure [4]. Doses greater than 0.4 units/min have been associated with bradycardia, decreased RV contractility, and decreased CO [4].

Epinephrine is a strong alpha-1 and beta-1 agonist [4]. Animal models have shown that epinephrine may be superior to dopamine in reducing the PVR/SVR ratio [4]. In human patients experiencing septic shock with RV failure, epinephrine administration has been shown to improve RV contractility and CO without adversely affecting PVR [4].

Phenylephrine, a selective alpha-1 agonist, has only vasopressor activity without impacting the contractility of the heart [4]. Therefore, by increasing SVR, it can improve perfusion to the RCA, but it also increases PVR, which can ultimately hinder RV function and decrease CO [4]. As such, it is not an ideal agent in the treatment of ARHF [17,19].

##### Anti-Arrhythmics

As previously discussed, RV failure can lead to the development of arrhythmias such as SVT, atrial fibrillation, and flutter. Bradyarrhythmias and ventricular arrhythmias are rare in patients with RV failure, except in cases of cardiac arrest [4]. The development of SVT is considered a negative prognostic marker in ARHF and is associated with right ventricular failure and increased mortality [37]. Patients with atrial tachyarrhythmias may benefit from electrical cardioversion, as the dysrhythmia can lead to worsening right ventricular cardiac output due to the loss of atrial kick [17], although large-scale studies on its use have yet to be studied in RV failure [4]. Amiodarone and digoxin are the leading choices for the restoration of sinus rhythm [34,37]. Radiofrequency ablation is another option for patients with recurrent SVT [1]. Rate control with beta blockers or calcium channel blockers can worsen cardiac output and is generally not recommended [17,34]. In patients with right-bundle branch block and RV dysfunction, AV pacing may enhance RV performance [4].

##### Oxygenation and Ventilation

It is imperative that patients with ARHF maintain oxygen saturation levels above 90–92% [4,10]. This can be achieved with supplemental oxygen or noninvasive ventilatory support. The goal is to avoid invasive mechanical support, as the use of sedatives and analgesics for intubation may potentially cause acute hypotension via nonselective vasodilation, leading to rapid deterioration of heart function and even cardiac arrest [1,10]. Further, more generation of positive pressure in the chest cavity can further decrease the venous return and RV stroke volume and may increase PVR by compressing pulmonary capillaries [7,8,17]. High-flow nasal cannulas and non-invasive pressure ventilation (NIV) should be considered for hypoxic patients prior to intubation [1,4]. 

Patients who are in ARHF and require endotracheal intubation should have their preload optimized with small fluid boluses and vasopressors as needed to maintain the mean arterial pressure (MAP) [8], and peri-intubation extracorporeal membrane oxygenation (ECMO) can be considered. The recommended induction agents are ketamine and etomidate, as they are more hemodynamically neutral [8]. Inhaled pulmonary vasodilators such as nitric oxide may be used prior to intubation and continued via the endotracheal tube to help improve PVR and oxygenation [8,17]. Ventilation parameters should minimize auto-PEEP, use low tidal volumes, avoid hypercapnia and hypoxia, and keep plateau pressures less than 30 mmHg to help maintain a low PVR [4,8,9,17]. Prone positioning may help patients with ARHF that are intubated by decreasing airway pressures and improving RV pressure overload parameters seen on echocardiography [4].

#### 3.4.2. Mechanical Circulatory Support (MCS)

For patients with acute right heart failure who are refractory to medical therapies, MCS may be a temporary measure, and options include extracorporeal membrane oxygenation (ECMO), right ventricular assist devices (RVAD), and pumpless lung assist devices. MCS can be particularly useful as a bridge while patients await heart-lung transplants [4,10].

##### Extracorporeal Membrane Oxygenation (ECMO)

Veno-arterial (VA) ECMO can be placed via peripheral or central cannulation and is effective at reducing right ventricular preload and pulmonary blood flow while maintaining oxygenation to essential organs [9]. VA-ECMO indirectly bypasses the right ventricle by providing retrograde flow via the femoral artery [8], thereby draining deoxygenated blood from the venous circulation and returning oxygenated blood to the arterial circulation [4]. When pulmonary vascular diseases are the predominant cause of ARHF, ECMO is the preferred MCS device as it avoids increases in pulmonary arterial pressures [12].

Potential complications of VA-ECMO include increased bleeding risk due to the need for anticoagulation, increased risk of infection, potential thrombus formation, and prolonged immobilization [3,10]. A recent meta-analysis investigated bivalirudin as an anticoagulant for patients on ECMO compared to heparin (the current standard of care) and found that bivalirudin showed decreased hospital mortality and thrombotic events (odd ratio 0.5) without any difference in major bleeding or ECMO duration [38]. Additionally, nafamostat mesylate (a synthetic serine protease inhibitor) was reviewed in a small sample of patients being treated with VA-ECMO as a regional anticoagulant and showed promising results of no adverse events of thrombosis and lower aPTT values measured at the patient compared to the ECMO site, with lower clinically significant bleeding compared to heparin [39].

In intubated patients with RV failure on VA-ECMO, patients are at risk for complications of prolonged sedation and mechanical ventilation on top of typical complications from ECMO [10]. However, there are case series that show promise in using VA-ECMO on awake patients, including improvement in renal failure with patients surviving until transplantation [10].

##### Right Ventricular Assist Devices (RVADs)

RVADs augment contractility and support the right ventricle. They can be implanted either surgically or percutaneously and are approved for use for up to 4 weeks (but have been used for several months) while awaiting improvement of RV function or more definitive treatment [3]. Surgical RVADs are implanted via cannulation of the right atrium, and blood is returned to the pulmonary artery using an extracorporeal pump [15,40]. For patients with PAH, RVAD should not be used, as it may predispose the patient to pulmonary hemorrhage [40]. 

Complications with RVADs include bleeding or thrombus formation [3]. As with other methods, RVADs do not seem to be a long-term option, and the definitive treatment is a transplant [3].

However, they have been shown to be beneficial in patients after RV infarction, cardiac surgery, left ventricular assist device implantation, and heart transplantation, as these mechanisms can sometimes lead to RV failure [4]. In patients with congenital heart disease leading to right ventricular failure (such as transposition of the great arteries), RVADs have demonstrated reductions in pulmonary arterial pressure and pulmonary capillary wedge pressure, leading to better functional cardiac outcomes and more successful hemodynamics following transplant [41,42]. In patients with biventricular failure, dual right and left-sided ventricular assisted devices, or VA-ECMO, are preferable to RVADs [30,40].

##### Pumpless Lung Assist Devices

Another option for mechanical circulatory support is a pumpless device that is inserted between the pulmonary arteries and left atrium [10]. A few case series have utilized this low-resistance oxygenating device in patients with pulmonary artery hypertension by connecting it between the main pulmonary artery and left atrium [10]. Patients require intubation for this procedure, so VA-ECMO may be a better strategy to avoid harmful sedating drugs [10].

## 4. Discussion

Right heart failure can be a challenging diagnosis to make in an acute setting in the emergency department, as it is difficult to distinguish right from left heart failure. As with most diagnoses, a thorough history and physical examination, taking care to note evidence of dyspnea, abdominal fullness, peripheral edema, elevated jugular venous pressure, and loud pulmonary component of heart sounds, may help guide the physician. Lab tests are fairly nonspecific; however, elevations in pro-BNP are suggestive of ARHF, and labs to assess signs of end organ damage can be a marker of the severity of disease and increased mortality. EKG, point-of-care ultrasound, and occasionally CT scans of the chest can give further diagnostic information regarding the hemodynamic status of the patient and potential causes for the exacerbation (see Table 1). There are some inciting factors, such as myocardial infarction or pulmonary embolism, which, if promptly recognized, have their own treatment algorithms that can improve the hemodynamic effects on the right ventricle. Overall, though, while treating the inciting factor, the provider must simultaneously balance preload, afterload, and heart contractility and further decide when to reach for the additional help of mechanical support.

The ultimate goal of caring for these patients is to choose the ideal medications to support them through the acute crisis, avoid an emergent need for transplant, and prevent further damage to the right heart (see Table 2). The vast majority of patients will present in a state of fluid overload necessitating diuretic therapy or ultrafiltration with renal replacement therapy. Due to the careful balance of volume status, some patients will require vasopressors, such as norepinephrine, to be able to tolerate the removal of volume while maintaining their cardiac output. Pulmonary dilators are a useful medication choice for individuals with known pulmonary hypertension driving the acute heart failure exacerbation, and the inhaled versions (such as nitric oxide) have more pulmonary specificity and less risk of hypotension. Efforts should be made by the physician to avoid intubating patients who are hypoxic and to instead override the pulmonary vasculature vasoconstriction by supplementing oxygen via noninvasive methods or a high-flow nasal cannula. Lastly, inotropic medications like dobutamine or levosimendan, which help improve the contractility of the right heart, may be necessary and can be used in conjunction with the other medications. There are some instances in which medications alone do not suffice, in which case mechanical adjuncts, such as VA-ECMO, are a suitable option.

Future directions should include randomized controlled trials aimed at studying different medication management strategies and short- and long-term outcomes. Additionally, emergency departments should develop treatment algorithms so that we may better streamline the care given to these patients and have firm guidelines for when to transfer the patient in the event of an acute right heart failure exacerbation.

## 5. Limitations

This study is a focused narrative literature review, specifically addressing acute decompensated right heart failure. It is not intended to be a systematic review, which would require adherence to the PRISMA statement. Our review aimed to prepare the emergency physician to manage patients presenting with acute right heart failure, with an emphasis on diagnosis and medical management. There does exist a spectrum of research that discusses in depth the risks and benefits of certain medications as they relate to specific diagnoses and takes a deeper dive into the nuances of managing pulmonary hypertension in acute right heart failure; however, that was outside the scope of this review. Additionally, we attempted to discuss mechanical support devices that would be encountered by in/familiar to an emergency provider; therefore, it is probable that other options exist, as this was not the primary purpose of this review. Lastly, multiple reviewers screened the included articles, and an inherent bias may exist.

## 6. Conclusions

Recognition of a patient in ARHF is essential, as treatment is complex and involves correcting underlying pathophysiology, optimizing RV preload, improving contractility, and decreasing afterload [9]. Management of volume status includes using diuretic therapy to improve cardiac function and reduce congestion and choosing the ideal vasodilators when necessary to reduce afterload. Medically optimizing perfusion and cardiac output with the use of inotropes and vasopressors and promptly treating hypoxia to avoid intubation is also necessary. Furthermore, the provider needs to consider mechanical circulatory support, such as VA-ECMO or RVADS, or lung transplantation in cases of refractory RHF despite maximal medical therapy [34].

## Figures and Tables

**Table 1:** Approach to acute right heart failure in the acute setting.

Diagnostic Tool	Findings
History	Dyspnea, anxiety, early satiety, tachycardia, tachypnea, hypoxemia, peripheral edema, relative hypotension, cool/clammy skin, abdominal fullness, or right upper quadrant tenderness
Physical exam	Elevated jugular venous pressure, ascites, or hepatojugular reflux, tricuspid regurgitation, a loud pulmonary component of the second heart sound (S2), fixed splitting of the S2, or an RV heave
Labs	- Signs of end-organ hypoperfusion with elevated lactate - Elevation in the nonspecific brain natriuretic peptides (BNP and NT-proBNP) - Hyponatremia, elevated CRP, and anemia have been associated with higher mortality
EKG	- Right atrial dilation, rightward axis, right ventricular hypertrophy, right bundle branch block, or arrhythmogenic right ventricular cardiomyopathy (ARVC)
POCUS	- Apical 4 chamber view: → right ventricular dilation and enlargement compared to the left ventricle - M-mode aligned with the tricuspid annulus → measurement of the tricuspid annular plane systolic excursion (TAPSE) <1.7 cm - Intraventricular septal flattening with a D-shaped LV - Plethoric inferior vena cava (diameter < 10 mm) without inspiratory collapse - Reduced fractional area change (<35%) - Left shift of the septum with paradoxical movement

**Table 2:** Medical management of acute right heart failure.

Target Mechanism	Therapy	Considerations
Preload reduction	- Loop diuretics (1st line) - Thiazide diuretics - Ultrafiltration	Avoid nitrates in RV infarcts
Afterload reduction	- Calcium channel blockers (nifedipine) - Prostanoids (epoprostenol, treprostinil, and iloprost) - Prostacyclin receptor agonist (selexipag) - Nitric oxide - Phosphodiesterase-5 (PDE5) inhibitors (sildenafil, tadalafil) - Soluble guanylate cyclase stimulator (riociguat)	Consider inhaled options for more pulmonary specificity
Inotropic Support	- Dobutamine (1st line) - Levosimendan, milrinone	
Vasopressors	- Norepinephrine (1st line) - Vasopressin, epinephrine	Avoid phenylephrine
Exacerbating Factors	- Hypoxia - Supplemental oxygen - Non-invasive ventilatory support - Arrhythmia - Amiodarone (1st line), digoxin - Cardioversion - Pulmonary Embolism - Consider thrombolytics - Infection - Broad-spectrum antibiotics	Avoid intubation if possible Avoid rate control with beta blockers and calcium channel blockers

## Supplementary Materials

The following supporting information can be downloaded at: https://www.mdpi.com/article/10.3390/jcm13030869/s1, Search Strategy.
