# Supplementary material for: Approach to Decompensated Right Heart Failure in the Acute Setting
Source: J Clin Med. 2024 Feb 2;13(3):869. doi: 10.3390/jcm13030869 (PMC10856072; doi:10.3390/jcm13030869)
Supplement: Supplementary file 1 [file jcm-13-00869-s001.zip › jcm-2838427 Supplementary Materials.pdf]

Supplementary Materials

Search Strategy

PubMed:

186

Search: **(right ventricle) AND ((decompensated) AND (right sided heart failure))** Filters: **Adult: 19+ years, English** Sort by: **Most Recent**

(("heart ventricles"[MeSH Terms] OR ("heart"[All Fields] AND "ventricles"[All Fields]) OR "heart ventricles"[All Fields] OR ("right"[All Fields] AND "ventricle"[All Fields]) OR "right ventricle"[All Fields]) AND ("heart ventricles"[MeSH Terms] OR ("heart"[All Fields] AND "ventricles"[All Fields]) OR "heart ventricles"[All Fields] OR ("right"[All Fields] AND "ventricle"[All Fields]) OR "right ventricle"[All Fields]) AND (("decompensate"[All Fields] OR "decompensated"[All Fields] OR "decompensating"[All Fields] OR "decompensation"[All Fields] OR "decompensations"[All Fields]) AND ("heart failure"[MeSH Terms] OR ("heart"[All Fields] AND "failure"[All Fields]) OR "heart failure"[All Fields] OR ("right"[All Fields] AND "sided"[All Fields] AND "heart"[All Fields] AND "failure"[All Fields]) OR "right sided heart failure"[All Fields]))) AND ((english[Filter]) AND (alladult[Filter]))

Translations

right ventricle: "heart ventricles"[MeSH Terms] OR ("heart"[All Fields] AND "ventricles"[All Fields]) OR "heart ventricles"[All Fields] OR ("right"[All Fields] AND "ventricle"[All Fields]) OR "right ventricle"[All Fields]

right ventricle: "heart ventricles"[MeSH Terms] OR ("heart"[All Fields] AND "ventricles"[All Fields]) OR "heart ventricles"[All Fields] OR ("right"[All Fields] AND "ventricle"[All Fields]) OR "right ventricle"[All Fields]

decompensated: "decompensate"[All Fields] OR "decompensated"[All Fields] OR "decompensating"[All Fields] OR "decompensation"[All Fields] OR "decompensations"[All Fields]

right sided heart failure: "heart failure"[MeSH Terms] OR ("heart"[All Fields] AND "failure"[All Fields]) OR "heart failure"[All Fields] OR ("right"[All Fields] AND "sided"[All Fields] AND "heart"[All Fields] AND "failure"[All Fields]) OR "right sided heart failure"[All Fields]

SCOPUS:

45

TITLE-ABS-KEY (decompensated AND right AND sided AND heart AND failure) AND (LIMIT-TO (SUBJAREA , "MEDI")) AND (LIMIT-TO (DOCTYPE , "ar")) AND (LIMIT-TO (LANGUAGE , "English")) AND (LIMIT-TO (EXACTKEYWORD , "Human"))
